# Rapid and continuous activity-dependent plasticity of olfactory sensory input

**DOI:** 10.1038/ncomms10729

**Published:** 2016-02-22

**Authors:** Claire E. J. Cheetham, Una Park, Leonardo Belluscio

**Affiliations:** 1 Developmental Neural Plasticity Unit, National Institute of Neurological Disorders and Stroke, National Institutes of Health, 35 Convent Drive, Bethesda, Maryland 20892, USA

## Abstract

Incorporation of new neurons enables plasticity and repair of circuits in the adult brain. Adult neurogenesis is a key feature of the mammalian olfactory system, with new olfactory sensory neurons (OSNs) wiring into highly organized olfactory bulb (OB) circuits throughout life. However, neither when new postnatally generated OSNs first form synapses nor whether OSNs retain the capacity for synaptogenesis once mature, is known. Therefore, how integration of adult-born OSNs may contribute to lifelong OB plasticity is unclear. Here, we use a combination of electron microscopy, optogenetic activation and *in vivo* time-lapse imaging to show that newly generated OSNs form highly dynamic synapses and are capable of eliciting robust stimulus-locked firing of neurons in the mouse OB. Furthermore, we demonstrate that mature OSN axons undergo continuous activity-dependent synaptic remodelling that persists into adulthood. OSN synaptogenesis, therefore, provides a sustained potential for OB plasticity and repair that is much faster than OSN replacement alone.

## Abstract (web summary)

New olfactory sensory neurons (OSNs) wire into olfactory bulb circuits throughout life. Here, the authors show that newly generated OSNs form highly dynamics synapses and can elicit functional responses in OB neurons, while mature OSNs retain a high level of activity-dependent synaptic reorganisation.

## Graphical abstract

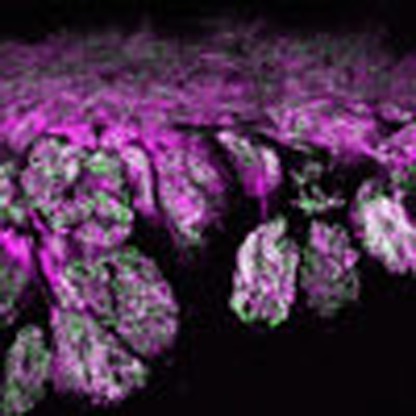

In the mammalian brain, most neurons are born *in utero* and assemble into functional circuits during late embryonic and early postnatal development. Hence, neuronal maturation and initial circuit formation are temporally related. In contrast, in the olfactory system, adult-born neurons are integrated into functional circuits throughout life^1^,^2^,^3^. In combination with its anatomy, the amenability of the olfactory bulb (OB) to both optical imaging and activity manipulation make it the ideal system in which to dissociate the cell autonomous and target-derived factors that regulate synaptogenesis in conjunction with sensory experience.

One of the regenerating neural populations of the olfactory system, the olfactory sensory neurons (OSNs), is located in the olfactory epithelium, and provides sensory input to the OB. OSNs take 7–8 days from terminal cell division to reach maturity, as defined by the onset of expression of olfactory marker protein (OMP)^4^,^5^. Each mature OSN expresses a single allele of one of several hundred odorant receptors, and OSNs expressing the same odorant receptor project their axons to the same glomerulus in the OB^6^,^7^,^8^, producing a highly organized anatomical odour map. Within the glomerulus, OSNs form excitatory synapses with both principal neurons and periglomerular interneurons^9^,^10^,^11^,^12^. However, how OSN synaptogenesis is governed by neuronal maturity remains an open question. During embryonic development, the onset of OMP expression coincides with formation of the first sensory synapses in the OB, at E14-15 (refs 13, 14, 15, 16). Hence, it is unclear whether maturation is a prerequisite for synaptogenesis, or vice versa. Furthermore, whether OSNs retain the capacity for synaptogenesis throughout their lifespan, or whether rewiring is instead effected purely by OSN turnover, is completely unknown. Understanding both when newborn neurons can initiate synaptogenesis, and whether any level of ongoing synaptogenesis is retained once neurons have matured, has profound implications for plasticity and repair of neural circuits.

Here, we used a genetic strategy to selectively label and manipulate immature and mature OSNs. Using electron microscopy, optogenetic photoactivation and *in vivo* multi-electrode recording, we demonstrate that OSNs still expressing immature markers form synapses and can evoke responses in OB neurons. We then use *in vivo* two-photon time-lapse imaging to show that mature OSNs retain a high level of activity-dependent synaptic reorganization, even in the adult OB.

## Results

### Immature OSNs form synapses with OB neurons

**Figure 1:**
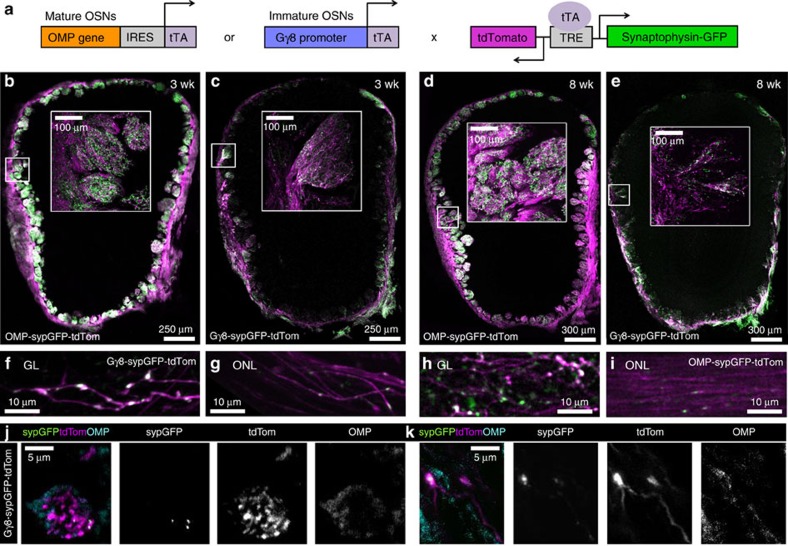
Specific labelling of immature and mature OSN axons in the OB. (**a**) Schematic representation illustrating generation of transgenic lines expressing tdTom and sypGFP specifically in either immature or mature OSNs using the tTA system. (**b**,**c**) Widefield fluorescence images of coronal sections through OBs of 3-week-old OMP-sypGFP-tdTom (**b**) and Gγ8-sypGFP-tdTom (**c**) mice. Inset: maximum intensity projections of confocal *z*-stacks for boxed regions. (**d**,**e**) As **b**,**c** but for 8-week-old mice. Note difference in scale bar for main images. (**f**,**g**) Two-photon images of immature OSN axons in glomerular (**f**) and outer nerve (**g**) layers of a Gγ8-sypGFP-tdTom mouse. (**h**,**i**) As **f**,**g** for an OMP-sypGFP-tdTom mouse. (**j**,**k**) Confocal images of anti-OMP staining in the GL of the OB of Gγ8-sypGFP-tdTom mice, showing little co-expression of OMP in Gγ8+ OSN axons.

To investigate the relationship between OSN maturity and synaptogenesis, we specifically labelled the axons and presynaptic terminals of either immature or mature OSNs using the tetracycline transactivator (tTA) system (Fig. 1a). tTA expression was driven either by Gγ8, which is expressed in immature, basally located OSNs^17^ (Supplementary Fig. 1A,B), or by OMP, an established marker for mature OSNs^18^. These driver lines were crossed with a tetO-synaptophysinGFP-tdTomato reporter line^19^, in which simultaneous expression of cytosolic tdTomato (tdTom) and GFP tagged-synaptophysin (sypGFP) are controlled by a tetracycline-responsive promoter, to generate Gγ8-sypGFP-tdTom and OMP-sypGFP-tdTom mice. In the olfactory epithelium of 8-week-old mice, we found that 98% of Gγ8+ OSNs expressing tdTom co-stained for GAP43, another widely used marker for immature OSNs^20^,^21^, while 6% also co-stained for OMP. Hence, we refer to Gγ8+ OSNs as ‘immature', while noting that a small subset of Gγ8+/OMP+ OSNs are likely at an intermediate stage, that is, in transition to maturity.

In 3-week-old OMP-sypGFP-tdTom mice, we observed mature OSNs densely innervating the OB, along with strong expression of sypGFP concentrated in the glomerular layer (GL) (Fig. 1b). Interestingly, immature OSN axons in Gγ8-sypGFP-tdTom mice also contained sypGFP and in some cases formed glomerular-like structures (Fig. 1c). In 8-week-old mice, expression patterns of both reporters were similar to those in younger mice, but there were fewer immature OSN axons innervating the OB (Fig. 1d,e), consistent with Gγ8 expression being limited to the regenerating OSN population at this age^17^. At higher resolution, expression of sypGFP in individual Gγ8+ axons was punctate and colocalized with axonal varicosities in the GL (Fig. 1f), indicative of synapse formation. In contrast, in the olfactory nerve layer, which lacks synapses, only smaller sypGFP puncta, which likely correspond to transport packets, were visible in the immature axons (Fig. 1g). Similarly, sypGFP expression in OMP-sypGFP-tdTom mice was punctate in the GL, with smaller transport packets in the olfactory nerve layer (Fig. 1h,i). We verified the specificity of sypGFP labelling of synaptic puncta by immunostaining OB sections from 8-week-old Gγ8-sypGFP-tdTom and OMP-sypGFP-tdTom mice and examining the GL. This showed that 97.5% of OSN presynaptic terminals in tdTomato-expressing axons, as identified by SV2 staining, contained sypGFP (Supplementary Fig. 1C). Hence, analysis of sypGFP puncta encompasses the vast majority of OSN presynaptic terminals. We also found >96% colocalization of sypGFP puncta with synaptophysin staining and 89% colocalization with apposed PSD-95 staining (Supplementary Fig. 1D–E and Supplementary Table 3). This indicates that the majority of sypGFP clusters form synapses. The remaining 11% of PSD-95-negative sypGFP clusters likely represent a combination of sypGFP transport packets, and synapses in the process of formation or elimination.

A small subset of Gγ8-tdTom OSNs co-stain for OMP, and hence are in transition to maturity. It was therefore important to determine what contribution these Gγ8+/OMP+ OSNs make to the axons innervating the OB in Gγ8-sypGFP-tdTom mice. We found 8.9% colocalization between tdTom and OMP, and 6.5% colocalization between sypGFP and OMP, in the GL of OB sections from Gγ8-sypGFP-tdTom mice immunostained for OMP (Fig. 1j,k and Supplementary Fig. 2). The slightly higher colocalization in axons in the GL compared with OSNs in the olfactory epithelium is not surprising, since the youngest Gγ8+ OSNs are likely to have short axons that have yet to reach the GL. Of the Gγ8+/OMP- axons in the GL of Gγ8-sypGFP-tdTom mice, 78% contained sypGFP puncta that colocalized with varicosities (*n*=71 axons from eight mice). The remaining 22% of Gγ8-tdTom axons that lack sypGFP puncta likely represent an earlier stage of OSN maturation, where OSN axons have entered the GL but have yet to form synapses. Together, these analyses confirm that while a small subset of Gγ8+ axons and the presynaptic terminals that they form are also OMP+, these are a minor contributor to the total population of glomerular axons expressing Gγ8-tTA-driven reporters.

**Figure 2:**
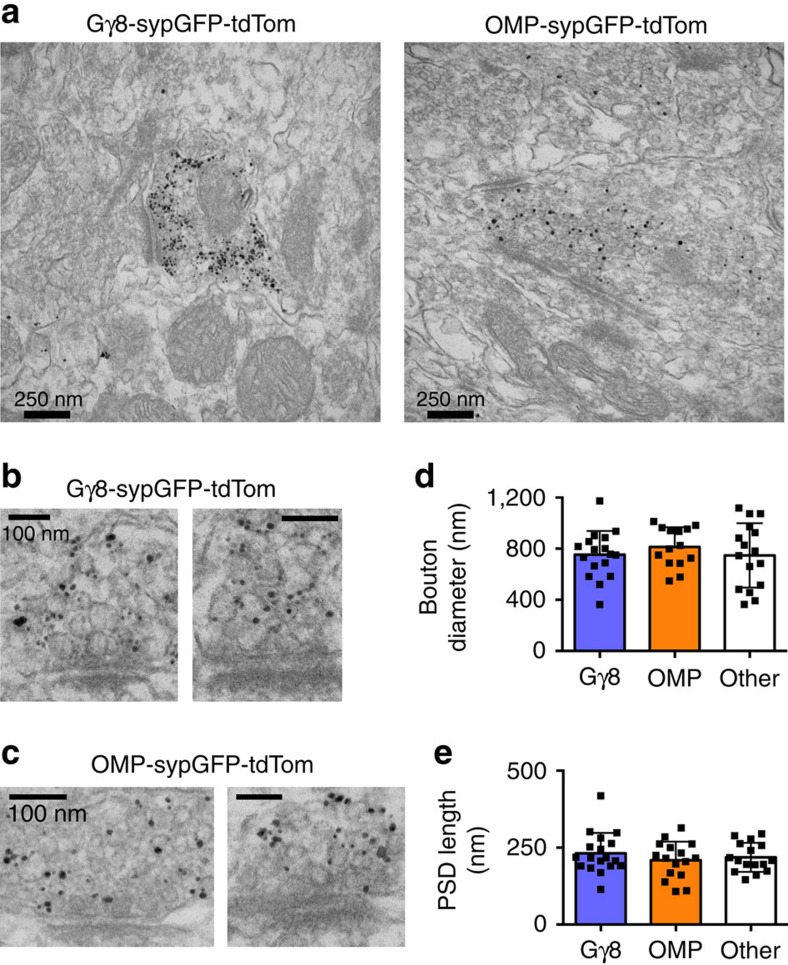
Immature OSN axons form synapses in the OB. (**a**) Low-magnification EM images from Gγ8-sypGFP-tdTom (left) and OMP-sypGFP-tdTom (right) mice demonstrating specific labelling of OSN presynaptic terminals with immunogold anti-GFP immunostaining. (**b**,**c**) Anti-GFP immunogold transmission electron micrographs of synapses formed by immature (**b**) or mature (**c**) OSN axons in the OB of 8-week-old mice. (**d**,**e**) No difference in maximum bouton diameter (**d**; *P*=0.36) or PSD length (**e**; *P*=0.55) between synapses formed by immature (*n*=18) or mature (*n*=16) OSNs or adjacent unlabelled synapses (‘other', *n*=16; one-way ANOVAs). Bar charts show mean±s.d.

To examine immature OSN axons in the GL of 8-week-old Gγ8-sypGFP-tdTom mice at the ultrastructural level, we performed immunogold anti-GFP staining and transmission electron microscopy. The anti-GFP antibody that we used detected 98.7% of sypGFP puncta, with no false positives at the light level (Supplementary Fig. 1F and Supplementary Table 3), and specifically labelled a subset of presynaptic boutons by electron microscopy (Fig. 2a). We found that immature OSN axons do form asymmetric synapses^22^: immunogold-labelled axonal boutons contained presynaptic vesicles apposed to a postsynaptic density across a synaptic cleft (Fig. 2b). Indeed, these synapses were structurally similar to synapses formed by both mature OSNs in OMP-sypGFP-tdTom mice, and adjacent unlabelled synapses (Fig. 2c–e). Although the identity of the presynaptic and postsynaptic structures forming these unlabelled synapses is unknown, the compartmentalization of glomerular neuropil^23^ makes it likely that they are also axodendritic OSN-OB neuron synapses. Hence, we concluded that immature OSNs can form synapses in the OB.

### Gγ8+ OSNs can evoke OB neuron firing

**Figure 3:**
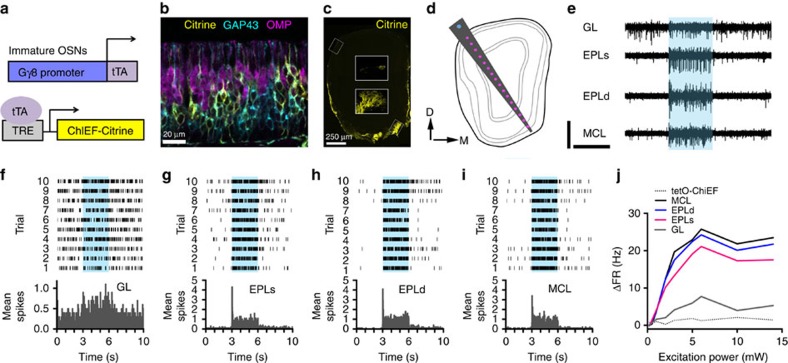
Optogenetic photoactivation of immature OSN axons evokes robust firing of OB neurons. (**a**) Schematic representation illustrating generation of transgenic mice expressing ChIEF-Citrine in immature OSNs using the tTA system. (**b**) Confocal image of coronal section of olfactory epithelium from P16 Gγ8-ChIEF-Citrine mouse showing colocalization with GAP43 (98.5%) and OMP (6.2%; *n*=1,106 Gγ8^+^ OSNs from three mice). 49.7% (1,106/2,225, *n*=3 mice) of GAP43^+^ neurons expressed Citrine. (**c**) Widefield fluorescence image of coronal OB section showing ChIEF-Citrine expression in olfactory nerve layer and GL of the same mouse as in **b**. Inset: maximum intensity projections of confocal *z*-stacks for boxed regions. (**d**) Schematic representation of multi-channel recording experiment. Optrode consisted of 16 electrodes (pink) with 100 μm spacing and an optical fibre (blue) terminating 200 μm above the most superficial electrode. D, dorsal; and M, medial. (**e**) Example traces from deepest four electrodes in a Gγ8-ChIEF-Citrine mouse showing robust multi-unit firing during photoactivation (blue band) in the GL, EPL (superficial and deep) and MCL. Scale bars, 10 μV, 1 s. (**f**–**i**) Raster plots and peri-stimulus time histograms (20 ms bins) for trials using an excitation power of 10 mW for the same mouse shown in **e**; traces correspond to trial 1. (**j**) Relationship between excitation power (473 nm) and mean change in firing rate (ΔFR) for neurons in GL, EPL and MCL in Gγ8-ChIEF-Citrine mice (*n*=5, mean values shown). GL *P*<0.001; EPLs *P*<0.001; EPLd *P*=0.003; MCL *P*=0.006; and Gγ8-ChIEF-Citrine versus tetO-ChIEF-Citrine, two-way ANOVA. tetO-ChIEF-Citrine data shown as mean values across all four electrodes (*n*=3).

For the synapses formed by immature OSNs to play a functional role in olfaction, they must be capable of transmitting information to OB neurons. To investigate this question, we generated a tetO-ChIEF-Citrine mouse line, which expresses the high-conductance, slowly inactivating channelrhodopsin variant ChIEF^24^ fused to the yellow fluorescent protein Citrine under control of a tetracycline-responsive promoter (Methods). By crossing this line to the Gγ8-tTA driver line, we produced specific expression of ChIEF-Citrine in immature OSNs (Fig. 3a–c and Supplementary Fig. 3A–C). In the olfactory epithelium of 3-week-old Gγ8-ChIEF-Citrine mice, close to the age at which the greatest number of Gγ8+ OSNs are present^25^, we found that 99% of Gγ8+ OSNs expressing Citrine colocalized with GAP43, while only 6% of Gγ8+ OSNs also expressed OMP (Supplementary Fig. 3C). Of these Gγ8+/OMP+ OSNs, 86% also expressed GAP43. Therefore, as in the Gγ8-sypGFP-tdTom line, the vast majority of Gγ8+ OSNs are immature and do not express OMP, while a small subset express both immature and mature markers (Gγ8+/GAP43+/OMP+), likely representing a transition stage. OSNs migrate apically from the basal lamina as they mature. We therefore determined the position of individual Gγ8+ and GAP43+ OSNs along the basal-apical axis of the olfactory epithelium as an approximate indicator of their maturity^26^. The ranges of positions along the basal-apical axis were identical for Gγ8+ and GAP43+ OSNs (both 5–79%). However, the distribution of Gγ8+ OSNs (median (inter-quartile range): 47 (36–58) %) was skewed towards slightly more apical positions than that of GAP43+ OSNs (40 (29–52) %; Supplementary Fig. 3D), indicating that Gγ8 is expressed slightly later in the maturation process than GAP43.

We then performed *in vivo* multi-electrode recording combined with optogenetic photoactivation in 3-week-old Gγ8-ChIEF-Citrine mice using a multi-channel optrode, which spanned the entire OB from the dorsolateral to the ventromedial side (Fig. 3d). We found that photoactivation with 473 nm light elicited robust time-locked firing of OB neurons for at least one electrode site in five out of six recorded Gγ8-ChIEF-Citrine mice. For the example shown in Fig. 3e–i, the change in multi-unit firing rate, calculated as the difference in mean firing rate during the 3 s light pulse relative to the preceding 3s baseline, was significant for the mitral cell layer (MCL) and both electrodes in the external plexiform layer (EPLs and EPLd). Across all six mice, photoactivation induced a significant increase in multi-unit firing frequency in the GL, external plexiform layer (EPLs and EPLd) and MCL of the OB (Fig. 3j and Supplementary Fig. 3E–H, Methods). Photoactivation-induced firing was excitation wavelength-specific (Supplementary Fig. 3I), and firing rate was dependent on excitation power (Fig. 3j and Supplementary Fig. 3E–H). In contrast, photoactivation in age-matched tetO-ChIEF-Citrine control mice did not evoke a significant change in firing rate in any OB layer (three mice, >150 total trials per mouse, Fig. 3j and Supplementary Fig. 3E–H). Hence, we concluded that immature OSNs are capable of evoking functional responses in OB neurons.

### Rapid turnover of OSN presynaptic terminals

We next asked whether the synapses formed by OSNs are stable or dynamic. The structural plasticity of primary sensory synapses has not previously been investigated. On the one hand, one might intuitively expect a primary sensory synapse, whose role is simply to relay information, to be relatively stable. On the other hand, synapse formation and elimination are known to be key plasticity mechanisms in many brain regions, including the OB^27^,^28^. If synaptogenesis is restricted solely to initial incorporation of an OSN into the glomerular circuit, then sensory input can be altered only by death and replacement of OSNs. However, if synapses can be formed and eliminated throughout the lifespan of an OSN, this plasticity would enable rapid and ongoing adaptation to changes in sensory experience.

**Figure 4:**
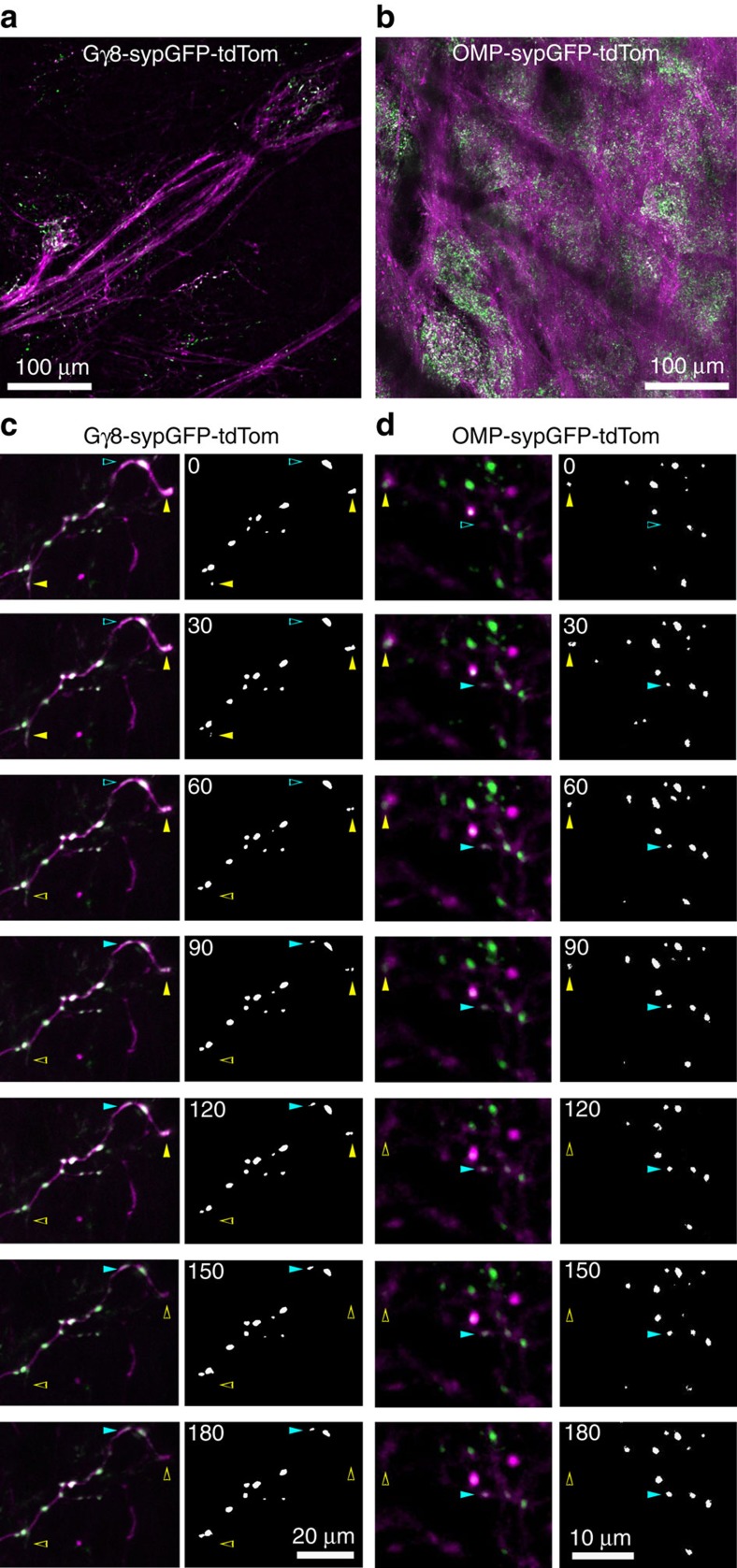
*In vivo* two-photon time-lapse imaging of OSN presynaptic terminals. (**a**,**b**) Maximum intensity projections of *in vivo* two-photon *z*-stacks showing the dorsal surface of the OB in 3-week-old Gγ8-sypGFP-tdTom (**a**) and OMP-sypGFP-tdTom (**b**) mice. (**c**) Maximum intensity projections of time-lapse images of an immature OSN axon. (**d**) Single optical sections of time-lapse images from an OMP-sypGFP-tdTom mouse. (**c**,**d**; left) Fluorescence images and (right) detected sypGFP puncta. Yellow arrowheads: puncta destined to be lost; cyan arrowheads: newly formed puncta.

To investigate this question, we developed a robust assay to quantify structural remodelling of synapses formed by OSNs. We implanted cranial windows over the dorsal surface of the OB of 3-week-old Gγ8-sypGFP-tdTom and OMP-sypGFP-tdTom mice (Fig. 4a,b), and used *in vivo* two-photon microscopy to repeatedly image presynaptic terminals formed by either immature or mature OSN axons throughout entire glomeruli (Supplementary Movies 1,2). Since maturation-related changes in reporter expression and OSN replacement could affect structural synaptic remodelling of OSN inputs over days to weeks^4^,^5^,^17^,^29^,^30^, we sought to minimize this contribution and thus confined our imaging experiments to a single 3 h session, during which each glomerulus was imaged at 30 min intervals. This approach enabled us to perform longitudinal three-dimensional (3D) tracking of sypGFP puncta in two-photon *z*-stacks, which are a proxy for OSN presynaptic terminals (Figs 2, 4c,d, Supplementary Fig. 1C–E, Supplementary Table 3 and Methods).

**Figure 5:**
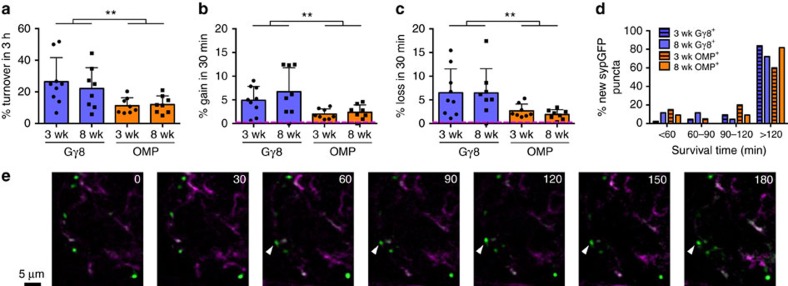
Rapid and continuous turnover of OSN presynaptic terminals. (**a**) Percentage turnover of sypGFP puncta over 3 h. *P*=0.002 Gγ8 versus OMP; *P*=0.23, 3-week old versus 8-week old, two-way ANOVA. (**b**) Mean percentage gain of new sypGFP puncta over 30 min. *P*=0.002 Gγ8 versus OMP, *P*=0.32, 3-week old versus 8-week old, two-way ANOVA. (**c**) Mean percentage loss of existing sypGFP puncta over 30 min. *P*=0.001 Gγ8 versus OMP; *P*=0.56, 3-week old versus 8-week old, two-way ANOVA. Magenta dashed lines in **b**,**c** indicate potential contribution of movement noise (Methods and Supplementary Fig. 4C). In **a**–**c**, mean±s.d. is shown. (**d**) Survival time of newly-formed sypGFP puncta. (**e**) Time-lapse image series of single optical sections from two-photon *z*-stack showing formation of new sypGFP punctum (arrowhead) at t60, which persists throughout the remainder of the imaging session (survival time >120 min).

We first examined turnover of OSN presynaptic terminals, which is a widely used metric for quantifying structural plasticity (Methods)^31^. Turnover comprises both formation of new presynaptic terminals and elimination of existing presynaptic terminals, thereby providing a measure of the total change during the 3 h imaging session. However, turnover does not imply direct replacement, as gain and loss can occur at completely independent locations. We found that in 3-week-old mice, immature OSN presynaptic terminals exhibited a very high level of turnover (26.5±15.3%; Fig. 5a), consistent with them being in the process of wiring into glomerular circuits. Surprisingly, however, mature OSN presynaptic terminals also underwent a significant level of turnover within 3 h (11.3±5.0%; Fig. 5a), although this turnover rate was 2.35-fold lower than that of immature OSN presynaptic terminals.

Since some glomeruli continue to mature into the second postnatal month^32^, we repeated our imaging experiments in 8-week-old mice to determine whether structural plasticity of OSN presynaptic terminals exhibits a developmental critical period. Interestingly, we found that turnover rates for both immature (22.2±13.2%) and mature (12.0±5.5%) OSN presynaptic terminals were very similar in 8-week-old mice to those in 3-week-old mice (Fig. 5a, see above) suggesting that this high level of structural plasticity continues into adulthood.

Although our analysis of turnover clearly demonstrates a high level of plasticity, this metric includes only those sypGFP puncta present during the first and/or last imaging session and so may not provide a complete picture of the dynamics of structural remodelling. Therefore, we utilized the information captured by imaging at 30 min intervals to assess the changes in OSN presynaptic terminals with greater temporal resolution. Rates of both formation (Fig. 5b) and elimination (Fig. 5c) of sypGFP puncta were higher for immature than mature OSNs, but again did not differ between 3-week-old and 8-week-old mice. Interestingly, formation of new sypGFP puncta and elimination of existing sypGFP puncta occurred at similar rates within each genotype (Fig. 5b,c; gain versus loss *P*=0.65, immature versus mature *P*<0.001, interaction *P*=0.82; two-way analysis of variance (ANOVA), data for 3-week- and 8-week-old mice pooled). Similarly, there was no systematic difference in the rates of sypGFP puncta gain and loss for individual glomeruli (*P*=0.62, paired *t*-test of all data in Fig. 5b,c), implying a steady-state level of presynaptic terminal turnover. Importantly, control experiments demonstrated that the high level of structural plasticity that we measured for OSN presynaptic terminals was not an artefact of our imaging protocol (Supplementary Fig. 4). Taken together, our data demonstrate rapid and continuous structural plasticity of sensory input to the OB into adulthood.

The precise timeline for the formation of a functional OSN synapse is unknown; however, studies of cultured hippocampal neurons suggest that synapse formation can occur in as little as 1 h (refs 33, 34, 35). Since we could not directly determine the lifetime of all of the sypGFP puncta that we imaged, we decided to track the survival of new sypGFP puncta that appeared during the first hour of our imaging session. We found that over 90% of newly formed sypGFP puncta across all experimental groups persisted for at least one hour, and over 75% persisted for two hours or longer (Fig. 5d,e). Hence, the majority of sypGFP puncta that were gained or lost during the 3 h imaging session had lifetimes greater than one hour, and were apposed to a PSD-95 punctum (Supplementary Fig. 1D and Supplementary Table 3), therefore they very likely correspond to synaptic structures. Furthermore, because the probability of stabilization of a newly formed sypGFP punctum was similar for immature and mature OSN axons, this suggests that the large differences in their structural dynamics arises from increased rates of formation and elimination of Gγ8-sypGFP puncta.

### Turnover of OSN presynaptic terminals is activity-dependent

**Figure 6:**
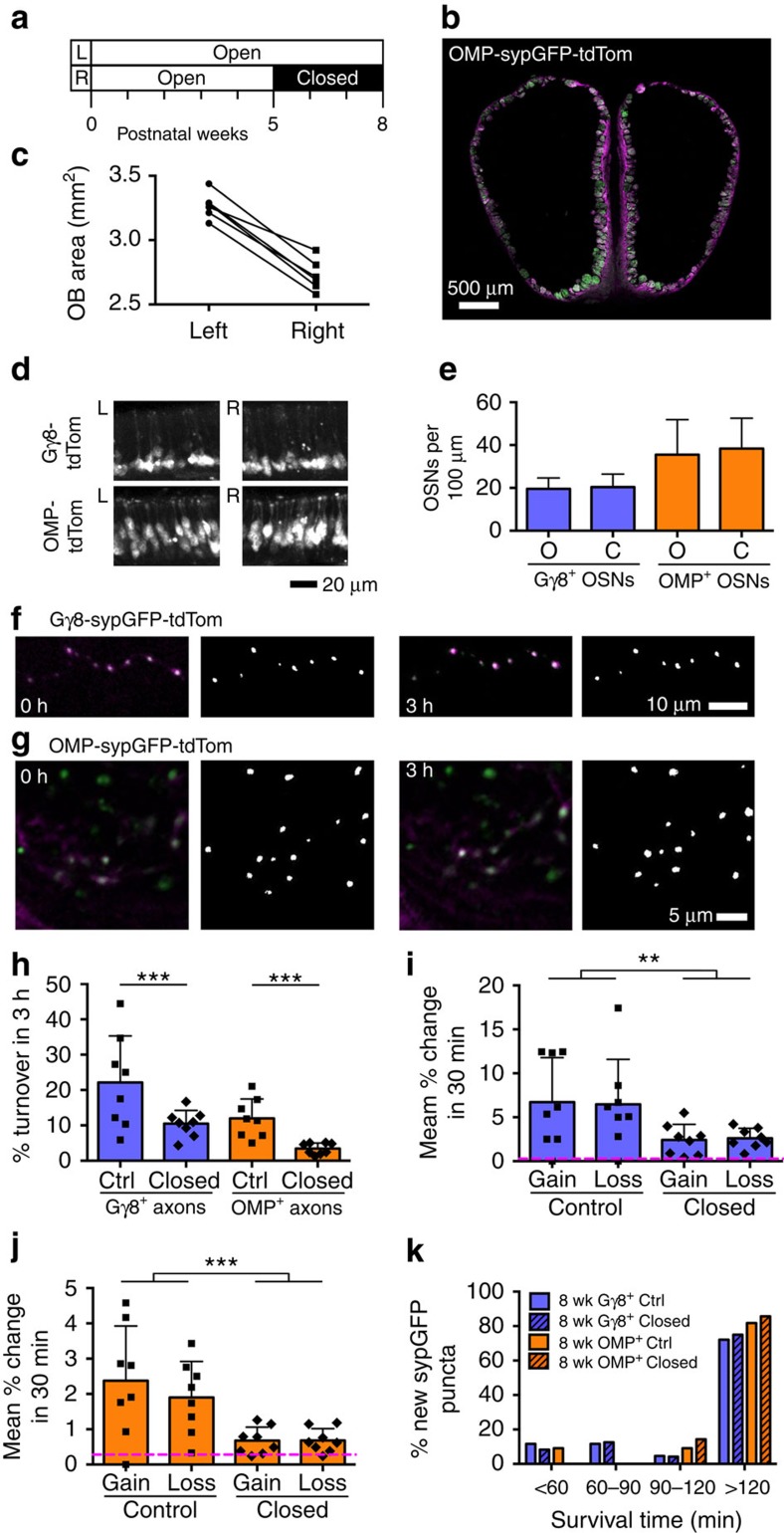
Turnover of OSN presynaptic terminals is strongly reduced by naris occlusion. (**a**) Timeline for naris occlusion. (**b**) Widefield image of OB coronal section from 8-week-old OMP-sypGFP-tdTom mouse following 3 weeks of naris occlusion, showing decreased OB size on the occluded (right) side. (**c**) Comparison of size of occluded (right) and open (left) OBs (*P*<0.001, paired *t*-test, *n*=6 mice). (**d**) Confocal images of left and right sides of septal olfactory epithelium in coronal sections. (**e**) No difference in density of Gγ8^+^ (*P*=0.87) or OMP^+^ (*P*=0.84) OSNs between open (O) and closed (C) nares. Two-way ANOVA; three mice/group. (**f**,**g**) Time-lapse images of Gγ8-sypGFP (**f**) and OMP-sypGFP (**g**) puncta showing stability over 3 h. (left) Acquired images and (right) detected sypGFP puncta. (**h**) Turnover of immature and mature OSN presynaptic terminals is strongly reduced in the ipsilateral OB following naris occlusion (*P*<0.001, effect of naris occlusion; *P*<0.001, effect of OSN maturity; *P*=0.046, interaction; two-way ANOVA). Ctrl: unoccluded mice. (**i**) Naris occlusion reduces gain and loss of immature OSN presynaptic terminals in the ipsilateral OB (*P*=0.005, effect of occlusion; *P*=0.98, gain versus loss; *P*=0.87, interaction; two-way ANOVA). (**j**) Naris occlusion reduces gain and loss of mature OSN presynaptic terminals in the ipsilateral OB (*P*<0.001, effect of occlusion; *P*=0.50, gain versus loss; *P*=0.48, interaction; two-way ANOVA). (**k**) Survival time of newly formed sypGFP puncta. Magenta dashed lines as Fig. 5b,c. Bar charts show mean±s.d.

Rapid and continuous turnover of OSN presynaptic terminals may enable optimization of glomerular circuitry to changes in sensory experience. Therefore, we next investigated the role of sensory activity in OSN presynaptic terminal turnover by performing unilateral naris occlusion in Gγ8-sypGFP-tdTom and OMP-sypGFP-tdTom mice (Fig. 6a). Naris occlusion was maintained for 3 weeks, ending at 8 weeks of age. We found that this naris occlusion protocol decreased ipsilateral OB size by 17±3% (Fig. 6b,c) but had no effect on the number of tdTom-expressing neurons in the olfactory epithelium of either line (Fig. 6d,e), or on total numbers of OSNs, proliferating or apoptotic cells in the olfactory epithelium (Supplementary Fig. 5). We also found no effect of naris occlusion on the percentage of each glomerulus that was occupied by OMP+ axons (control OB 71±20; closed OB 77±18; *P*=0.084, *t*-test) or OMP-sypGFP puncta (control 44±26; closed 41±24; *P*=0.44 *t*-test; *n*=61 glomeruli from five mice per group).

In contrast, turnover of OSN presynaptic terminals was strongly down-regulated for both immature (2.11-fold reduction) and mature (3.48-fold reduction) OSN axons in the OB located ipsilateral to the occluded naris (Fig. 6f–h). We found that both formation of new puncta and loss of existing puncta were significantly reduced following naris occlusion for both classes of axons (Fig. 6i,j). Interestingly, however, the survival time of newly formed sypGFP puncta was similar in control and naris-occluded mice for both immature and mature OSNs (Fig. 6k). This indicates that while the rates of synaptogenesis and synapse elimination are markedly reduced by naris occlusion, the temporal behaviour of those few sypGFP puncta that are still formed is unaffected by activity blockade. Together, these data show that OSNs maintain a continuous capacity for activity-dependent synaptic remodelling that may be exploited to re-tune olfactory input based upon recent sensory experience.

## Discussion

We show here that immature, Gγ8-expressing OSNs form asymmetric synapses (Fig. 2), and that optogenetic photoactivation of immature OSN axons could evoke robust, stimulus-locked firing of OB neurons (Fig. 3). Furthermore, presynaptic terminals formed by immature OSNs were highly dynamic, undergoing >20% turnover in 3 h (Figs 4, 5). Surprisingly, mature, OMP-expressing OSN axons retained a high level of presynaptic terminal remodelling, with >10% turnover in 3 h in both juvenile and adult mice (Figs 4, 5). This plasticity was reduced 3.5-fold following 3 weeks of naris occlusion (Fig. 6). Hence, our data support two major novel findings. First, we provide evidence that while still expressing immature markers, OSNs form synapses and can evoke responses in OB neurons. Second, we show that OSNs not only undergo rapid synaptogenesis as they integrate into adult OB circuits but also maintain a high level of activity-dependent structural plasticity once they have matured. This provides a strategy for continuous re-optimization of OB circuitry, which acts on a much faster timescale than production of new OSNs.

Our EM data show that immature OSNs form synapses with very similar structural properties to those formed by their mature counterparts. This finding is consistent with a previous electrophysiological study showing that the presynaptic properties of OSN inputs remain constant as they mature^36^. However, it was not previously recognized that many of the newly-formed synapses were in fact made by immature OSNs.

We then asked whether the synapses formed by immature OSNs are functional, and found that optogenetic photoactivation of Gγ8-expressing axons evoked robust firing of OB neurons. Although there is a slight possibility that ChIEF-mediated depolarization of Gγ8+ axons is transmitted to neighbouring mature OSN axons via ephaptic coupling^37^, the most parsimonious explanation for optogenetically evoked responses in Gγ8-ChIEF-Citrine mice is that newly generated Gγ8+ OSNs can form functional synapses, which release glutamate and thereby depolarize their postsynaptic targets. 6.5% of Gγ8-sypGFP puncta colocalize with OMP; hence, a small proportion of the Gγ8+ OSNs that form synapses are already in transition to maturity^38^, and we cannot definitively rule out the possibility that these Gγ8+/OMP+ synapses could account for the optogenetically evoked firing of OB neurons. Nevertheless, our data suggest that OSNs begin to play a functional role earlier in their maturation process than previously thought. Accordingly, a recent study provided strong evidence that OSNs express odorant receptors two days prior to the onset of OMP expression^26^. Hence, OSNs that still express immature markers may be capable of detecting odours, which in combination with our data, suggests a new and previously unappreciated role for newly generated OSNs in olfactory function.

It has been suggested that synapse formation by OSN axons triggers OMP expression^15^,^16^, based on the similar timing of initial axodendritic synaptogenesis in the GL and OMP expression onset during embryonic development^13^,^14^,^15^,^16^. Our data support this model, and suggest that initial synapse formation by immature OSNs may promote their final maturation and survival. In agreement with our findings, analysis of OSNs labelled by electroporation found branched axons containing punctate sypGFP in the GL as early as 4 days post-terminal cell division^39^. In contrast, a recent study using Ascl-1-driven labelling of basal stem cells indicated that OSN axons do not enter the GL until 8 days after terminal cell division, concluding that synapse formation is not required for OMP expression^26^. These studies raise interesting questions about gauging OSN maturation with respect to different developmental markers and stages of axon development, further highlighting the need for investigation of OSN synaptogenesis. Definitively determining the temporal relationship between synapse formation and OMP expression in individual OSNs will require multi-day time-lapse imaging of sparsely-labelled OSN axons expressing distinct reporters under the control of both immature and mature OSN-specific promoters.

It is perhaps surprising that a primary sensory synapse, in this case providing the sole source of sensory input to the main OB, would undergo rapid and continuous structural remodelling. Indeed, the level of synapse turnover exhibited by mature OSN presynaptic terminals is significantly greater than that of excitatory synapses in other regions of the adult brain^27^. Nevertheless, similarly rapid structural remodelling has been reported for both dendritic protrusions in juvenile rat cortical circuits^40^ and presynaptic terminals of retinotectal axons in zebrafish larvae^41^. The magnitude of OSN presynaptic terminal turnover does however raise the issue of how sensory input to glomerular circuits is maintained. The answer may lie in regulating the balance of this turnover: first, the rates of formation and elimination of sypGFP puncta are very similar, suggesting that the number of synapses per glomerulus remains relatively stable. Second, a recurring theme in other sensory brain regions is the existence of subpopulations of synaptic structures with different lifetimes^41^,^42^,^43^. If this were also true of mature OSN presynaptic terminals, then a subpopulation of stable synapses could maintain a baseline level of sensory input to glomeruli, while their more dynamic counterparts serve other functions.

So what is the purpose of rapid structural remodelling of OSN synapses? Synapse turnover clearly plays an essential role during circuit formation (and in the case of the OB, incorporation of newborn neurons into existing circuits) by enabling selection, refinement and error correction. Hence, transient pre- or post-synaptic structures may represent those that fail to locate a synaptic partner, or form inappropriate connections that are rapidly eliminated. This may explain why immature OSN presynaptic terminals are formed and eliminated more rapidly than their mature counterparts (Figs 4, 5). Alternatively, these transient synaptic structures may represent short-lived synaptic contacts that temporarily contribute to network function^43^, or play other roles such as promoting axon branch stabilization^41^. Whatever the role of transient synaptic structures, ongoing synapse formation and elimination endows OB circuits with a plasticity potential that can be harnessed when needed, such as during learning^44^,^45^ or in response to altered experience^27^.

Importantly, turnover of both immature and mature OSN presynaptic terminals was strongly reduced following naris occlusion even in 8-week-old mice. In marked contrast, motility of dendritic protrusions in somatosensory cortex was dependent on sensory experience only during a brief critical period during the second postnatal week^40^. Our naris occlusion experiments were designed to assess the effects of a long-term reduction in olfactory input. OSN presynaptic terminal turnover is likely to have reached a new steady-state level after three weeks of naris occlusion. Hence, the reduction in both formation and loss of OSN presynaptic terminals is likely due to a combination of loss of odour-evoked activity and longer-term compensatory plasticity. Although it remains to be determined how rapidly OSN presynaptic terminal turnover is affected by naris occlusion, it is clear that the marked reduction in OSN presynaptic terminal turnover elicited by 3 weeks of naris occlusion was not a consequence of changes at the level of the olfactory epithelium, as we found no effect of naris occlusion on numbers of immature or mature OSNs, or proliferating or apoptotic cells. This is in agreement with a recent study, which found no change in epithelium thickness, total number of OSNs or number of OMP+ OSNs at time points 3–28 days post naris occlusion in adult mice^46^. Hence, a transient effect of naris occlusion on OSN survival is also unlikely.

It is possible that the level of OSN presynaptic terminal turnover differs between anaesthetised and awake mice. Our imaging experiments were performed in mice lightly anaesthetized with sevoflurane, a GABA_A_ receptor agonist^47^, and dexmedetomidine, an α_2_-adrenergic receptor agonist. OSN presynaptic terminals are glutamatergic, and there is no reported evidence that they express either GABA_A_ or α_2_-adrenergic receptors; hence, the anaesthetic agents used here are unlikely to have a direct pharmacological effect on OSN synapses. We used sevoflurane specifically because it is a weak odorant^48^; nevertheless, odour-evoked firing rates of mitral/tufted cells are significantly lower in sevoflurane-anaesthetised than awake mice^49^. Whether sevoflurane-mediated suppression of odour-evoked activity might affect OSN presynaptic terminal turnover is unclear, but given that chronic naris occlusion dramatically reduced OSN presynaptic terminal turnover, sevoflurane is more likely to suppress than to accelerate the turnover of OSN presynaptic terminals. Future studies could directly assess the rate of OSN presynaptic terminal turnover in awake mice.

Why invoke synapse turnover rather than other forms of plasticity? OSN presynaptic terminals have a uniformly very high presynaptic release probability, which does not differ between newly formed and long-standing synapses, or with the identity of the postsynaptic target^36^,^50^. Hence, OSN presynaptic terminals appear to have a limited functional dynamic range, and consequently, structural remodelling may be necessary in order to effect significant plasticity at the level of OB sensory input. In addition, synapse formation and elimination can rapidly alter the connectivity of glomerular circuits. OSNs are known to provide monosynaptic input to both principal neurons and at least two classes of periglomerular interneurons^9^,^10^,^11^,^12^. Therefore, rapid structural plasticity at the level of OSN inputs provides a powerful mechanism for dynamic regulation of excitatory-inhibitory balance, which is key to both information processing and plasticity in the OB^51^,^52^. Although OSN regeneration is vital for ongoing maintenance of olfactory function and recovery from damage to the olfactory epithelium or olfactory nerve^53^, it does not enable rapid or fine-scale tuning of OB circuitry. For example, a recent study found that the synaptic output of OSNs, measured at the resolution of individual glomeruli, increased as a result of fear learning^54^. These changes occurred within 3 days, too fast to be accounted for by incorporation of newborn OSNs^54^. However, activity-dependent formation and elimination of OSN synapses could be used to adjust glomerular connectivity and thus provide a cellular mechanism for this type of experience-induced plasticity^54^.

During development, neurons form highly organized networks that are optimized to the input that they receive during critical or sensitive periods of heightened plasticity^55^. Adult-born neurons must achieve a similar outcome, but in the non-permissive environment of the adult brain^56^. Importantly, our data suggest that the maturity of the neuron rather than the age of the animal is the critical determinant of OSN plasticity. Since this inherent plasticity is naturally derived from olfactory epithelial stem cells, it is possible that other stem cell-derived neurons may possess similar capacities that can be engineered to provide a viable therapeutic strategy for neural circuit repair in the adult brain.

## Methods

### Experimental animals

All animal procedures conformed to National Institutes of Health guidelines and were approved by the National Institute of Neurological Disorders and Stroke Institutional Animal Care and Use Committee. Mice were bred in-house and were maintained on a 12 h light/dark cycle with food and water *ad libitum*.

The tetO-ChIEF-Citrine line, was generated from pCAGGS-I-oChIEF-mCitrine-I-WPRE (7.7kb; Roger Tsien, UCSD)^24^, which contains the coding sequence for mammalian-optimized ChIEF fused to the yellow fluorescent protein Citrine. To place the ChIEF-Citrine coding region (1.8 kb) under the control of the tetracycline-responsive promoter TRE, it was subcloned into the pTre-tight vector (2.6 kb, Clontech). The resulting pTre-tight-ChIEF-Citrine vector was linearized and injected into the pronuclei of zygotes from FVB/N mice at the National Institute of Mental Health Transgenic Core Facility (Bethesda, MD). Transgenic founders were screened with two primer pairs that amplified either a ∼300 bp DNA fragment within the ChIEF coding region (CTTTCTGATGTCGCCGTG and GGCATCTACACCTTCTTC ), or a 660 bp DNA fragment within the Citrine coding region (GACGTAAACGGCCACAAGT and TCGAGTCGACCTACTTGTACAGCTCGTCCA ). We obtained four founder animals from 24 offspring from the pronuclear injection and chose to maintain the line with the strongest ChIEF-Citrine expression (based on Citrine fluorescence).

Generation of other transgenic lines has been described previously: Gγ8-tTA (ref. 57); OMP-IRES-tTA (ref. 58); CaMKIIα-tTA and TRE-Bi-SG-T (described here as tetO-sypGFP-tdTom) (ref. 19). Gγ8-tTA and OMP-tTA mice were obtained from Nick Ryba (NIDCD) and CaMKIIα-tTA (#7004) and TRE-Bi-SG-T (#12345) from The Jackson Laboratory (Bar Harbour, ME). Experimental animals were: Gγ8-ChIEF-Citrine [*Gγ8-tTA*^*+/−*^*/tetO-ChIEF-Citrine*^*+/−*^]; tetO-ChIEF-Citrine [*tetO-ChIEF-Citrine*^*+/−*^]; Gγ8-sypGFP-tdTom [*Gγ8-tTA*^*+/−*^*/tetO-sypGFP-tdTom*^*+/−*^]; OMP-sypGFP-tdTom [*OMP-tTA*^*+/−*^*/tetO-sypGFP-tdTom*^*+/−*^]; and CaMKIIα-sypGFP-tdTom [*CaMKIIα-tTA*^*+/−*^*/tetO-sypGFP-tdTom*^*+/−*^]. All mice were of mixed 129 × C57BL/6J background. The ages of mice in different experimental groups are detailed in the relevant sections of the Materials and Methods and the Results. Three-week-old mice were male and female and 8-week-old mice were male.

### Genotyping

OMP-sypGFP-tdTom, Gγ8-sypGFP-tdTom, CaMKIIα-sypGFP-tdTom and Gγ8-ChIEF-Citrine pups were detected by visualization of fluorescence in the nose and OB of P0-2 pups under epifluorescence illumination. Genotypes of a subset of these mice, and of littermates for breeding and control experiments, were determined by PCR using the primers listed in Supplementary Table 1.

### Perfusion and immunohistochemistry

Naive or previously imaged or recorded mice were deeply anaesthetized with 200 mg kg^−1^ ketamine and 20 mg kg^−1^ xylazine (both Vedco, Saint Joseph, MO) and transcardially perfused with ice-cold PBS followed by 4% paraformaldehyde (PFA). OBs and/or olfactory epithelia were dissected out and post-fixed overnight in 4% PFA at 4 °C, cryopreserved in 30% sucrose in PBS for 24 h at 4 °C, embedded in 10% gelatin, fixed/cryopreserved in 15% sucrose/2% PFA in PBS overnight and flash frozen in 2-methyl butane on dry ice. Coronal sections were cut using a CM3050S cryostat (Leica Microsystems, Buffalo Grove, IL) at 35-40 μm for OBs and 50 μm for olfactory epithelia, and either mounted directly in Vectashield (Vector Laboratories, Burlingame, CA) or stored at −80 °C. For immunohistochemistry, free-floating sections were incubated for 20 min in 1% sodium borohydride, blocked in 5% horse serum/0.1% gelatin/0.5% Triton-X100 for 1 h, and incubated with primary antibody in 3% horse serum/0.2% Triton-X100 for 48 h at 4 °C, then secondary antibody for 90 min at room temperature, before mounting in Vectashield. Primary antibodies are listed in Supplementary Table 2. The SV2 antibody, developed by K.M. Buckley, was obtained from the Developmental Studies Hybridoma Bank (DSHB), created by the NICHD of the NIH and maintained at The University of Iowa, Department of Biology, Iowa City, IA 52242. Secondary antibodies were Alexa Fluor-647-conjugated antibodies directed against the relevant species (Jackson Immunoresearch, West Grove, PA), all at 1:400.

Images were acquired using a Leica TCS SP5 microscope equipped with HCX PLAN FLUOTAR × 10/0.30 NA air and HCX PL APO × 40/1.25 NA oil immersion objectives and spectral detectors. Widefield images had a pixel size of 0.72 × 0.72 μm. Confocal image voxel size was 0.18 × 0.18 × 0.45 μm for OB (except colocalization with synaptophysin, GFP, PSD-95 and OMP, where voxel size was 0.14 × 0.14 × 0.2 μm) and 0.36 × 0.36 × 1 μm for olfactory epithelium images (all 1,024 × 1,024). For SV2, synaptophysin, GFP and PSD-95 colocalization analysis (Supplementary Fig. 1 and Supplementary Table 3), puncta were detected as for *in vivo* images (see below). A correlation *P*<0.0001 along line intensity profiles in sypGFP and anti-syp or anti-GFP channels was required for classification of a punctum as colocalized. Colocalization analysis of SV2 and sypGFP was similar, but was restricted to SV2 puncta in tdTom-expressing OSN axons. PSD-95 apposition was defined as peaks in sypGFP and PSD-95 intensity profiles within 0.5 μm. Colocalization of Gγ8-driven tdTom or sypGFP with OMP (Fig. 1j,k, Supplementary Fig. 2) was determined by quantifying pixels containing both OMP and tdTom fluorescence as a proportion of the number of tdTom+ or GFP+ pixels following Otsu thresholding^59^.

### OSN counts and position in the olfactory epithelium

For quantification of OSN density and colocalization with GAP43 or OMP, ∼1 mm of septal olfactory epithelium from each naris was analysed in each of three coronal sections (25, 50 and 75% along the anterior-posterior axis) for each mouse. To quantify the position of OSNs in the olfactory epithelium, we measured the distance of the centre of the OSN soma from the border with the basal lamina, as a proportion of the distance from the basal lamina to the apical surface of the epithelium. Positions of Gγ8+ and GAP43+ OSNs were measured in coronal sections 50% along the anterior-posterior axis for six Gγ8-ChIEF-Citrine mice immunostained for GAP43.

### Immunogold staining and electron microscopy

Eight-week-old Gγ8-sypGFP-tdTom or OMP-sypGFP-tdTom mice were deeply anaesthetized with 200 mg kg^−1^ ketamine and 20 mg kg^−1^ xylazine and transcardially perfused with 4% PFA/ 0.1% glutaraldehyde (Electron Microscopy Sciences, Hatfield, PA). Dissected OBs were post-fixed for 30 min in 4% PFA/0.1% glutaraldehyde before sectioning. 100 μm thick vibratome sections were blocked and permeabilized in 5% horse serum/0.1% saponin in PBS for 1 h prior, then stained with rabbit anti-GFP primary antibody (#632592, Clontech, Mountain View, CA) diluted 1:100 for 2 h followed by goat anti-rabbit-nanogold secondary antibody (10 nm gold particles, Fab' fragment, #2004, Nanoprobes, Yaphank, NY) diluted 1:200 for 1 h, and fixed in 2% glutaraldehyde in PBS for 1-5 days at 4 °C after staining. Sections were then washed six times in dH_2_O, and silver enhancement (HQ silver enhancement kit, Nanoprobes) was carried out in the dark using a red safety light. Sections were developed for 10 min, then washed repeatedly in dH_2_O for 5 min. EM processing was as described previously^60^. Briefly, sections were treated with osmium tetroxide followed by uranyl acetate, dehydrated and embedded in epoxy resin. Sections 60-nm thick were cut onto grids, and images of the GL were acquired using a 1200-EX II transmission electron microscope (Jeol, Peabody, MA). The GL was identified in two ways: (1) the olfactory nerve layer was clearly identifiable by the presence of dense axonal bundles; and (2) OSNs form synapses only in the GL^61^.

### *In vivo* optrode recording

Multi-channel recording was performed as described previously^62^. Briefly, recordings were made in ∼3-week-old (P16-19) Gγ8-ChIEF-Citrine (*n*=6) and tetO-ChIEF-Citrine (*n*=3) control mice under urethane anaesthesia. The skull was thinned over the left OB, a small craniotomy was made and a 16-channel silicon-based optrode (A1 × 16–5 mm-100-703-OA16LP, NeuroNexus Technologies, Ann Arbor, MI) was inserted (Fig. 3d). The 100 μm core-diameter optical fibre was connected to a 473 nm solid-state variable-power (100 mW maximum) laser (LaserGlow Technologies, Toronto, Canada) via a patch cord. The optogenetic stimulation protocol was designed to mimic commonly used odour stimulation paradigms, using a 3 s duration light pulse and varying the stimulation intensity. The inter-trial interval was ⩾20 s. Approximate laminar positions of individual electrodes were determined from local field potentials; we refer to the deepest four electrodes as GL, EPLs, EPLd and MCL. Raw data were high-pass filtered at 500 Hz to isolate multi-unit activity, and spikes with amplitudes >5 × root-mean-square noise were sorted. Change in firing rate (ΔFR) was calculated by subtracting the number of spikes in a 3 s window immediately prior to photoactivation from the number of spikes during the 3 s light pulse, and converting to Hz. A significant ΔFR was defined as having *P*<0.00625 (0.05 Bonferroni-corrected for eight different stimulus intensities tested for each mouse) for an unpaired two-tailed *t*-test comparing FR during the stimulus with FR during the 3 s baseline window^63^.

To test excitation wavelength specificity, photoactivation was performed using a metal halide lamp and 470/40 (excitation power 32 mW; 0.31 mW mm^−2^) and 562/40 (excitation power 65 mW; 0.63 mW mm^−2^) band-pass excitation filters (Leica MZ16F; Supplementary Fig. 3I). A Master-8 (A.M.P.I., Jerusalem, Israel) controlled the timing of light pulses. Optical fibre output power was measured using a FieldMaster GS power meter (Coherent, Santa Clara, CA), and ranged from 0.5 to 14 mW (Fig. 3j and Supplementary Fig. 3E–H), within the range used previously for optical fibre-mediated ChR2 stimulation of OSN axons *in vivo*^64^,^65^,^66^. Data in Fig. 3j and Supplementary Fig. 3E–H are from 10 to 12 trials at each excitation power for each mouse. Data in Supplementary Fig. 3I are from 15 to 20 trials at each excitation wavelength for each mouse.

### *In vivo* two-photon imaging

Experimental animals were P21-22 (3-week old) and P56-60 (8-week old) Gγ8-sypGFP-tdTom or OMP-sypGFP-tdTom mice (all *n*=6 per group), and P56-60 CaMKIIα-sypGFP-tdTom mice (*n*=3). Mice were anaesthetized with isoflurane (4% induction; 1.5–2% maintenance) in O_2_ (1 l min^−1^) and received a subcutaneous injection of ketoprofen (5 mg kg^−1^). The pinch withdrawal reflex was monitored throughout surgery. A small craniotomy (∼1.0 mm diameter) was made over the right OB. Special care was taken not to damage the underlying dura mater. The dura was rinsed with sterile buffer (125 mM NaCl, 5 mM KCl, 10 mM D-glucose, 10 mM HEPES, 2 mM CaCl_2_, 2 mM MgSO_4_ and pH 7.4), and the exposed brain was covered with a custom-cut number 1 coverglass, which was sealed with superglue (Loctite 411, All-Spec Industries, Wilmington, NC) and then dental cement (Orthojet, Lang Dental, Wheeling, IL). The surface of the skull was covered with superglue and dental cement, and a 0.1 g metal bar was attached to the skull for imaging. For P56-60 CaMKIIα-sypGFP-tdTom mice, a 1 mm diameter craniotomy was instead made over primary somatosensory cortex.

For imaging, mice were lightly anaesthetized with 0.5 mg kg^−1^ dexmedetomidine (s.c.) in lactated Ringer's solution and 1% sevoflurane in O_2_ (1 l min^−1^). To maintain stable anaesthesia during imaging experiments (3.5–4 h duration), a 20% top-up of the initial dose of dexdomitor was given up to every hour for 3-week-old mice, and a single top-up of 25% of the initial dose was given after 1.5–2 h for 8-week-old mice. Mice were placed under the microscope in a custom-made stereotaxic device via the metal bar attached to the skull in a fixed orientation relative to the objective lens. Mice were imaged with a Leica TCS SP5 microscope using a HCX APO × 20/1.00 NA water-immersion objective lens (Leica Microsystems) and a Chameleon Vision II IR laser (Coherent) mode-locked at 910 nm. The laser beam was expanded to fill the 2 cm diameter back aperture of the objective. Point spread functions measured by imaging 100 nm fluorescent beads embedded in 1% agarose were 0.46±0.05 μm in *xy* and 2.28±0.23 μm in *z* (mean±s.d., *n*=10 beads).

For each mouse, an initial low-resolution image (400 nm per pixel in *xy* and 2 μm in *z*) was acquired, and from this, regions of interest (ROIs), typically containing a single glomerulus were selected for high-resolution imaging (0.90 nm per pixel in *xy* and 1 μm in *z* (all 1,024 × 1,024 pixels)). Each ROI was imaged every 30 min for 3 h, that is, a total of 7 time points. For each ROI, identical laser power and detector gain settings were used for each time point. ROIs were 60–180 μm in *z*. GFP emission was collected at 500–550 nm, and tdTom emission at >600 nm. Control experiments imaging single fluorochromes showed no cross-talk between channels. Fluorescence levels were adjusted to maximize use of the dynamic range, without saturation. In CaMKIIα-sypGFP-tdTom mice, ROIs of the same size as for OB imaging were selected at random.

In a subset of imaged mice, a short-term time-lapse image series was acquired to assess the possible contribution of motion artefacts arising from breathing and/or blood vessel pulsation to measurements of synapse turnover (Figs 5b,c, 6h,i and Supplementary Fig. 4). This image series consisted of four high-resolution images of entire glomeruli acquired as described above, but at 3 min intervals.

We performed two additional control experiments. First, to exclude the possibility that imaging at 30 min intervals induced structural plasticity, we performed a control imaging experiment in two P21-22 OMP-sypGFP-tdTom mice (Supplementary Fig. 4D). Surgery and the first imaging time point were exactly as described above. Mice were then removed from the stereotaxic apparatus, received 1 mg kg^−1^ atipamezole and a bolus of warmed lactated Ringer's solution and were returned to their home cage (containing their mother and littermates) with a heat pad. These mice were ambulatory within 5 min of atipamezole administration, and remained in their home cage for ∼2.5 h, where they were observed to explore their cage and interact normally with littermates. They were then lightly anaesthetized with 0.5 mg kg^−1^ dexdomitor (s.c.) in lactated Ringer's solution and 1% sevoflurane in O_2_ (1 l min^−1^) for a second imaging session, which occurred exactly 3 h after the first. Second, in two 8-week-old OMP-sypGFP-tdTom mice, we used a thinned skull preparation instead of performing a craniotomy (Supplementary Fig. 4E). A ∼1 mm diameter region over the right OB was thinned with a microsurgical blade to a final thickness of 20–30 μm. Imaging was then performed exactly as for mice with implanted cranial windows, but using PBS as the immersion medium for the objective lens.

### Naris occlusion

Gγ8-sypGFP-tdTom (*n*=5) and OMP-sypGFP-tdTom (*n*=6) mice underwent naris occlusion at 5 weeks of age by inserting a small plug into the right naris under brief isoflurane anaesthesia. Naris occlusion continued for 3 weeks, such that mice were 8 weeks old on the day of imaging (Fig. 6a).

After 3 weeks of naris occlusion, and prior to cranial window implantation, the integrity of the block was confirmed by the absence of bubbles in a drop of water placed over the occluded naris. The effectiveness of blockade was further confirmed in perfusion-fixed tissue from each mouse by reduced size of the right OB (Fig. 6b,c) and in a subset of mice, by reduced OB tyrosine hydroxylase immunoreactivity in the right OB^67^. OB size was measured for three Gγ8-sypGFP-tdTom and three OMP-sypGFP-tdTom mice, as the surface area of the left and right OB for three coronal sections per mouse, located ∼25, 50 and 75% along the anterior-posterior axis. The mean of these values for each mouse is shown in Fig. 6c. A two-way ANOVA showed no effect of genotype on OB size (effect of genotype, *P*=0.63; effect of occlusion, *P*<0.001; interaction, *P*=0.37). Because of their density and highly branched structure, it was not possible to directly quantify the effect of naris occlusion on the number of OSN axons innervating individual glomeruli. We instead used the percentage of each glomerulus occupied by OMP-tdTomato axons or OMP-sypGFP puncta, as proxy measures. The percentage of the glomerulus occupied by tdTomato or GFP signal was measured in single confocal optical sections as described previously^46^.

### Two-photon image analysis

All image analysis was performed in Fiji^68^. Data analysis was performed blind to the genotype, age and experimental condition (naris occlusion versus control) of the mouse. Custom-written macros were used to generate an automated, unbiased image-processing pipeline. For each region of interest, images at all time points were first realigned using the ‘Correct 3D Drift' plugin, which permits only linear translations of the entire image in *x*, *y* and/or *z*. The tdTomato channel was used as the reference for realignment. Images were median filtered (two pixel radius), and sypGFP puncta were detected in 3D using an à trous wavelet decomposition at three length scales^69^ and a hard threshold *t*=s.d.√(2ln(number of pixels)^70^ was applied. ‘3D Objects Counter' was then used to index and label sypGFP puncta for manual tracking over time. The centres of individual sypGFP puncta analysed changed by <0.5 μm between time points, relative to fiducial points along the axon. The variance in quantification of OSN presynaptic terminal turnover was 1.42±1.48% (mean±s.d.) between different analysers, and hence could not explain the differences between experimental groups that we report. Turnover was defined as ([*n*_gain_+*n*_lost_]/[*n*_t0_+*n*_t3h_]) and reported as a percentage; survival fraction (SF) was defined as SF_t_=*n*_t_/*n*_0h_ (ref. 31). Analysis of turnover, gain, loss and survival fraction included only those sypGFP puncta present for at least two consecutive time points, in order to minimize any possible contribution of sypGFP transport packets. We typically analysed one to two glomeruli per mouse. We confirmed that the density of sypGFP puncta, which was higher in OMP-sypGFP-tdTom than in Gγ8-sypGFP-tdTom mice, did not affect detection of individual puncta or quantification of their turnover (Supplementary Fig. 4F–H). Linear manipulations of image brightness and contrast were performed for display purposes only.

We performed an additional test to determine whether formation and elimination of sypGFP puncta was independent of the threshold used for object segmentation. For a subset of images, we repeated the automatic object detection, but using a twofold lower threshold, and tracked the detected sypGFP puncta over the 3 h imaging session. We then made a pairwise comparison with the original tracking data for individual sypGFP puncta in these images. We found that the measurements of turnover, gain and loss were identical between the two thresholds. Indeed, the only minor discrepancy between the two data sets was that 1/218 puncta was lost 30 min later with the lower threshold. Hence, sypGFP puncta dynamics were largely independent of the intensity threshold employed during automatic object detection, and the one discrepancy that we identified makes a minimal contribution given the large magnitude differences between experimental groups that we report (Figs 5a-c,6h–j).

### Statistics

All data were normally distributed with equal variance. Data are described as mean±s.d. unless otherwise stated, and were compared using Student's *t*-test or one- or two-way ANOVA using Prism 6 (GraphPad Software, La Jolla, CA).

## 

## Additional information

**How to cite this article:** Cheetham, C. E. J. *et al*. Rapid and continuous activity-dependent plasticity of olfactory sensory input. *Nat. Commun.* 7:10729 doi: 10.1038/ncomms10729 (2016).

## Supplementary Material

Supplementary Information Supplementary Figures 1-5, Supplementary Tables 1-3 and Supplementary References.

Supplementary Movie 1 Immature OSN axons innervating a single glomerulus in a juvenile Gγ8-sypGFP-tdTom mouse.

Supplementary Movie 2 Mature OSN axons innervating a single glomerulus in a juvenile OMP-sypGFP-tdTom mouse.
