# Supplementary material for: Rapid and continuous activity-dependent plasticity of olfactory sensory input
Source: Nat Commun. 2016 Feb 22;7:10729. doi: 10.1038/ncomms10729 (PMC4764868; doi:10.1038/ncomms10729)
Supplement: Supplementary Information — Supplementary Figures 1-5, Supplementary Tables 1-3 and Supplementary References. [file ncomms10729-s1.pdf]

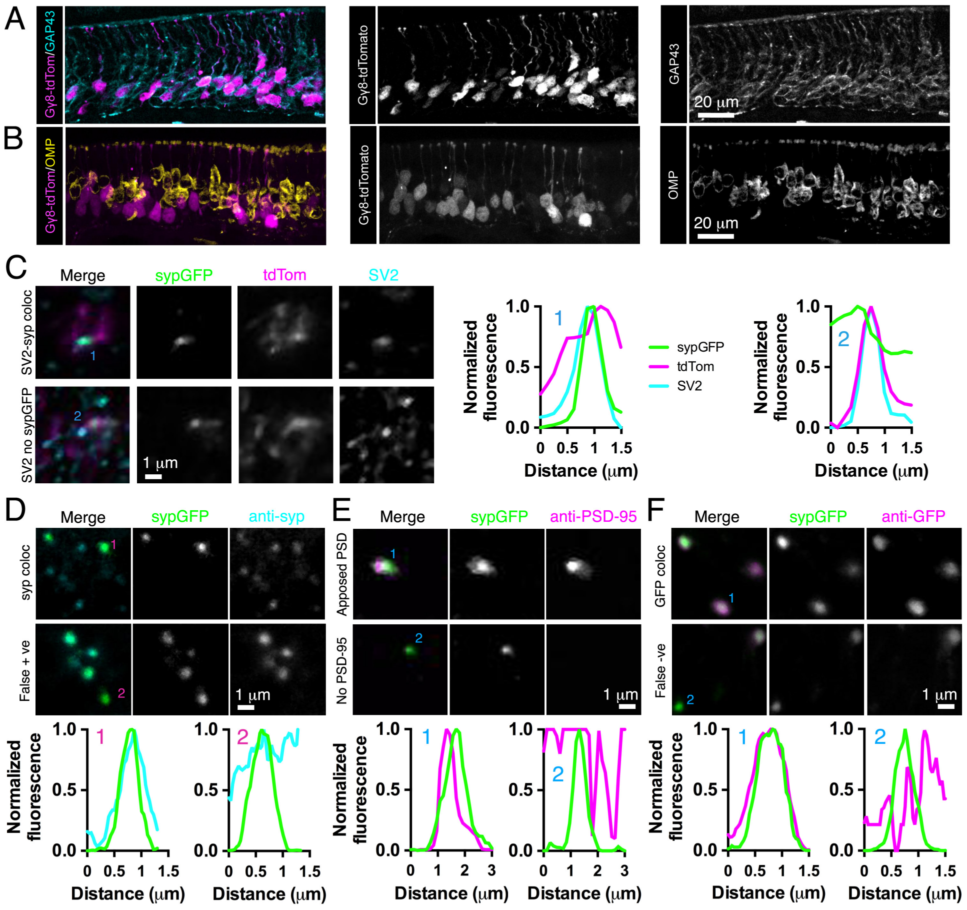

Supplementary Figure 1: Validation of labeling specificity of immature OSNs and presynaptic terminals.

(A) Confocal images of septal olfactory epithelium of an adult Gγ8-sypGFP-tdTom mouse showing colocalization of OSNs expressing tdTom with GAP43. **(B)** Confocal images of the septal olfactory epithelium of an adult Gγ8-sypGFP-tdTom mouse showing little colocalization with OMP. Colocalization was analyzed for Gγ8^{+} OSNs with GAP43 (n = 653 Gγ8+ OSNs from two mice) or OMP (n = 566 Gγ8^{+} OSNs from two mice). **(C)** Colocalization of sypGFP with OSN presynaptic terminals marked by SV2 staining in tdTomato-expressing axons. n = 129 SV2 puncta in OMP-sypGFP-tdTom OB tissue and 110 SV2 puncta in Gγ8-sypGFP-tdTom OB tissue. Left column shows merged images. Numbers in blue correspond to fluorescence intensity profiles shown to the right. (1) Colocalization of sypGFP and anti-SV2 staining. (2) Example of an SV2 punctum with no sypGFP. **(D)** Colocalization of sypGFP with anti-syp immunostaining in a Gγ8-sypGFP-tdTom mouse. Left column shows merged images. Numbers in magenta correspond to fluorescence intensity profiles shown below. (1) Colocalization of sypGFP and anti-syp staining. (2) Example of a false positive sypGFP punctum with no anti-syp staining. **(E)** Colocalization of sypGFP puncta with anti-PSD-95 immunostaining in a Gγ8-sypGFP-tdTom mouse. Left column shows merged images. Numbers in blue correspond to

fluorescence intensity profiles shown below. (1) Colocalization of sypGFP with anti-PSD-95 staining. (2) Example of a false positive sypGFP punctum with no anti-PSD-95 staining. **(F)** Colocalization of sypGFP with anti-GFP staining in a Gγ8-sypGFP mouse. Left column shows merged images. Numbers in blue correspond to fluorescence intensity profiles shown below. (1) Colocalization of sypGFP with anti-GFP staining. (2) Example of a false negative sypGFP punctum with no anti-GFP staining.

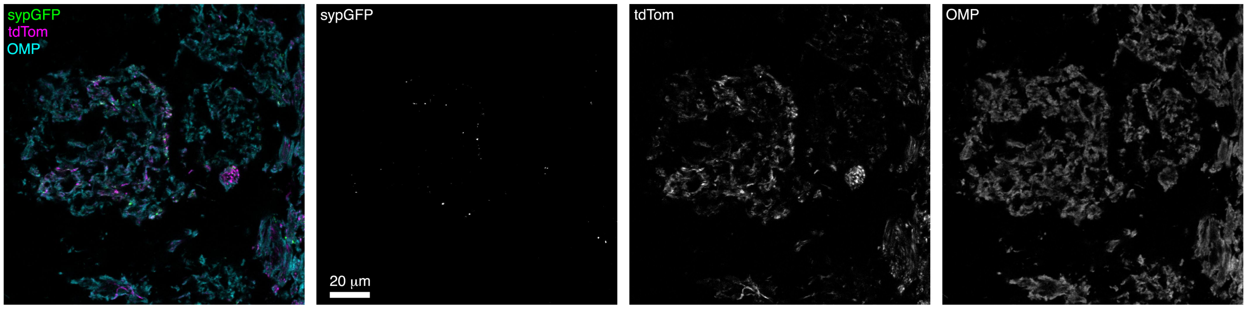

Supplementary Figure 2: Lower magnification confocal images of anti-OMP staining in the glomerular layer of the OB of a Gγ8-sypGFP-tdTom mouse.
Lower magnification (relative to Fig. 1J,K) confocal images of anti-OMP staining in the glomerular layer of the OB of a Gγ8-sypGFP-tdTom mouse, showing little co-expression of OMP in Gγ8+ OSN axons.

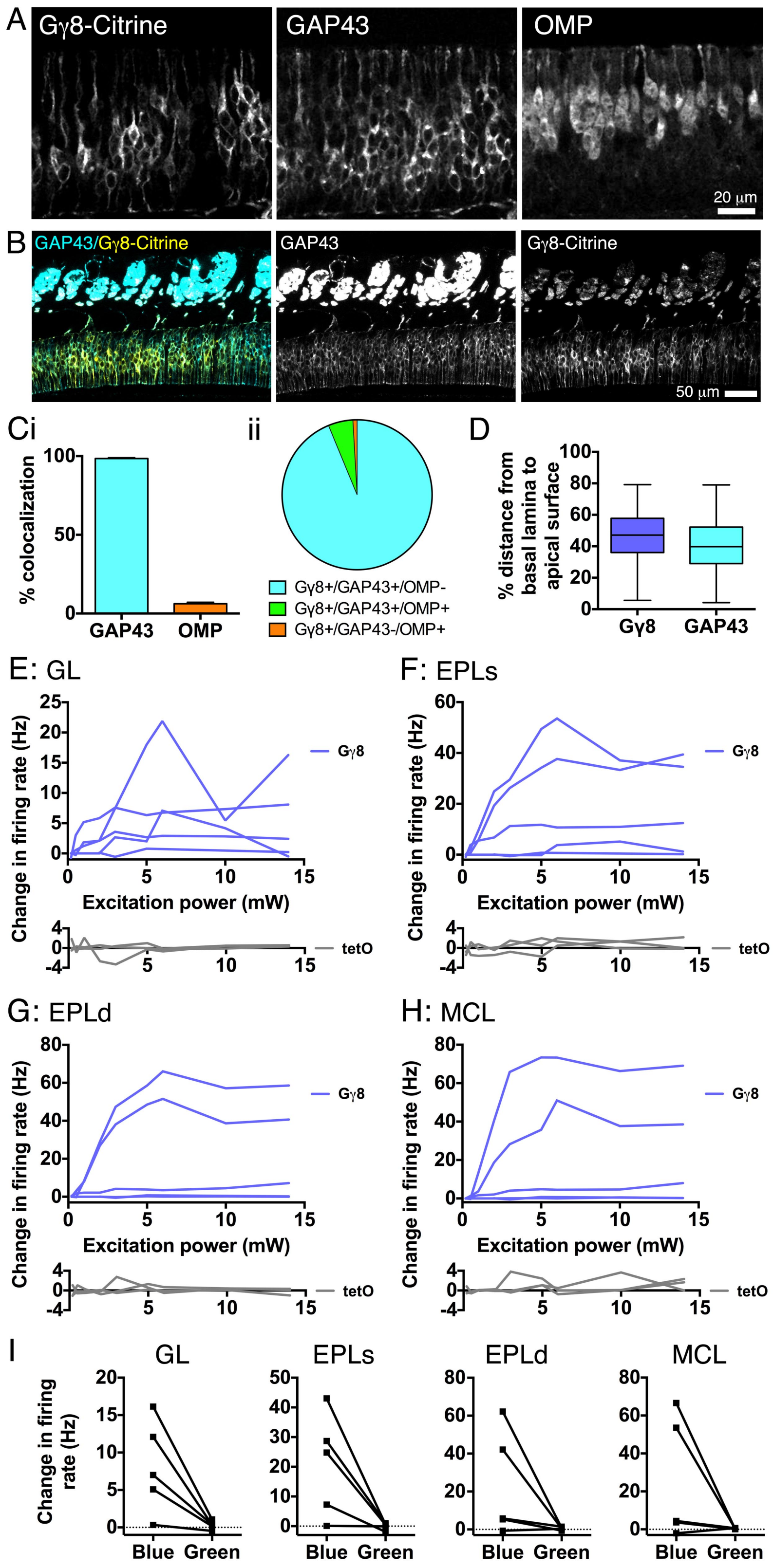

A
Gγ8-Citrine
GAP43
OMP
20 μm
B
GAP43/Gγ8-Citrine
GAP43
Gγ8-Citrine
50 μm
Ci
% colocalization
GAP43
OMP
ii
Gγ8+/GAP43+/OMP-
Gγ8+/GAP43+/OMP+
Gγ8+/GAP43-/OMP+
D
% distance from basal lamina to apical surface
Gγ8
GAP43
E: GL
F: EPLs
G: EPLd
H: MCL
Change in firing rate (Hz)
Excitation power (mW)
Gγ8
tetO
I
GL
EPLs
EPLd
MCL
Change in firing rate (Hz)
Blue
Green

Supplementary Figure 3: Optogenetic photoactivation is wavelength- and excitation power-dependent in Gγ8-ChIEF-Citrine mice.

(A) Confocal images of septal olfactory epithelium of a P16 Gγ8-ChIEF-Citrine mouse. Individual channels, corresponding to the merged image shown in Fig. 3B, are shown. **(B)** Confocal images of septal olfactory epithelium of a Gγ8-ChIEF-Citrine mouse co-stained for GAP43, illustrating the dense axon bundles formed by OSN axons as soon as they enter the lamina propria (top). **(C)** Quantification of overlap between immature and mature markers in Gγ8-ChIEF-Citrine olfactory epithelium. (i) Percentage colocalization of Gγ8+ OSNs with GAP43 and OMP immunostaining. Data shown as mean ± s.d. (ii) Proportion of Gγ8+ OSNs expressing GAP43 and/or OMP (n = 1115 Gγ8+ OSNs from 3 mice). **(D)** Box-and-whisker plot of position of OSNs expressing Gγ8-Citrine (n = 883) or GAP43 (n = 1969) in the olfactory epithelium. Box shows median and quartiles; whiskers extend to minimum and maximum values. $P < 0.001$, Kolmogorov-Smirnov test. **(E - H)** Dependence of change in multi-unit firing rate on excitation power density for five individual Gγ8-ChIEF-Citrine mice. Overall, 5/6 mice exhibited a significant increase in firing rate in response to 473nm photoactivation ($P < 0.00625$, t-test vs. baseline firing rate). Data are shown for 4/5 of the mice that showed responses and the one non-responder mice. No power curve was obtained for the fifth 'responder' mouse. Lower panels show the absence of a light-induced increase in firing rate in any layer in tetO-ChIEF-Citrine mice ($P > 0.05$, t-test vs. baseline for all layers, n = 3 mice). **(I)** Quantification of excitation wavelength dependence of change in multi-unit firing rate in response to widefield photoactivation of the dorsal surface of the OB in Gγ8-ChIEF-Citrine mice (n = 5). Blue (450 – 490 nm) light stimulation elicited increased firing rates across multiple layers whereas green (542 – 582 nm) light stimulation did not alter firing rate in any layer ($P = 0.001$, effect of wavelength, 2-way ANOVA). Data are values for individual mice.

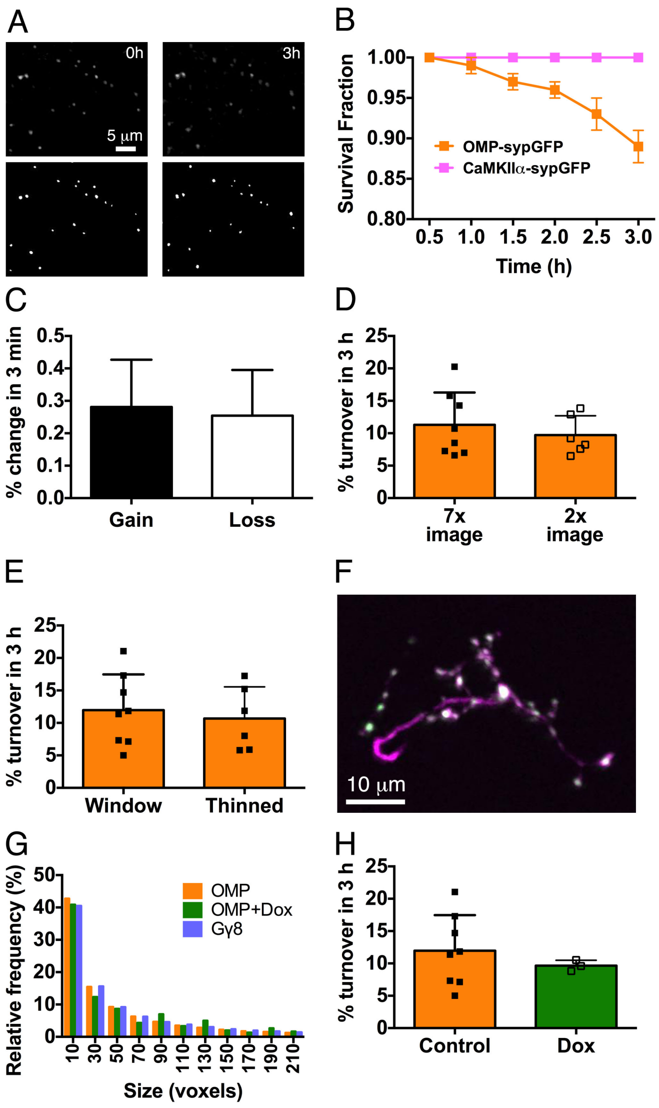

Supplementary Figure 4: Controls for *in vivo* 2-photon imaging protocol.
(A) Time-lapse imaging of CaMKIIα-sypGFP puncta. Upper panels show sypGFP fluorescence; lower panels show detected sypGFP puncta, for the first (0h) and last (3h) time points. Note that all puncta present at 0h are still present at 3h. **(B)** Comparison of survival fraction of OMP-sypGFP puncta in the OB (n = 8 glomeruli from 6 mice) and CamKIIα-sypGFP puncta in layer 2 of primary somatosensory cortex (n = 3 regions of interest from 2 mice) in 8 wk-old mice. No loss of CaMKIIα-sypGFP puncta was observed during 3h imaging sessions. Data are expressed as survival fraction to enable comparison with previous studies[1,2]. Data shown as mean ± s.d. **(C)** % gain and loss of sypGFP puncta that could be attributable to movement artifacts. Values were determined by acquiring four z-stacks through each glomerulus at 3 min intervals. Gain and loss were quantified as for 30 min imaging intervals. The value for mean % change is shown as a magenta dashed line in Fig. 5B,C and Fig. 6I,J. Data shown as mean ± s.d. **(D)** Repeated imaging of OSN presynaptic terminals does not alter turnover of OSN presynaptic

terminals. Turnover rates for presynaptic terminals of 3 wk-old OMP-sypGFP-tdTom mice are similar whether imaged with our standard protocol (7x image; 7 images acquired at 30 min intervals, anesthetized throughout) or imaged only twice with a 3 h interval between, awake in the home cage for ~2.5 h between imaging sessions (2x image). P = 0.51, t-test; n = 8 glomeruli from 6 mice (7x image) and n = 6 glomeruli from 2 mice (2x image). Note that these data do not negate the use of 30 min imaging intervals, which are required to capture structural dynamics on a timescale shorter than 3h that could be missed by quantifying turnover alone. Data shown as mean ± s.d. **(E)** Turnover rates for presynaptic terminals of 8 wk-old OMP-sypGFP-tdTom mice are similar whether imaged through a cranial window (as for all other data in this study) or through thinned skull. P = 0.66, t-test; n = 8 glomeruli from 6 mice (cranial window) and n = 6 glomeruli from 2 mice (thinned skull). Data shown as mean ± s.d. **(F)** Maximum intensity projection showing sparse labeling of mature OSN axons and presynaptic terminals in an 8 wk-old OMP-sypGFP-tdTom mouse raised on Dox food until 2 weeks prior to the imaging session (Methods). **(G)** Histogram of sizes of detected sypGFP puncta showing no difference between mature OSNs (OMP), sparsely labeled mature OSNs (OMP+Dox), and immature OSNs (Gγ8; P = 0.94, one-way ANOVA on Ranks). Histogram bin size is 20 voxels. **(H)** Turnover rates for mature OSN presynaptic terminals in 8 wk-old OMP-sypGFP-tdTom mice are similar whether they are densely labeled (Control) or sparsely labeled (Dox). P = 0.50, t-test; n = 8 glomeruli from 6 mice (Control) and n = 3 glomeruli from 2 mice (Dox). Data shown as mean ± s.d.

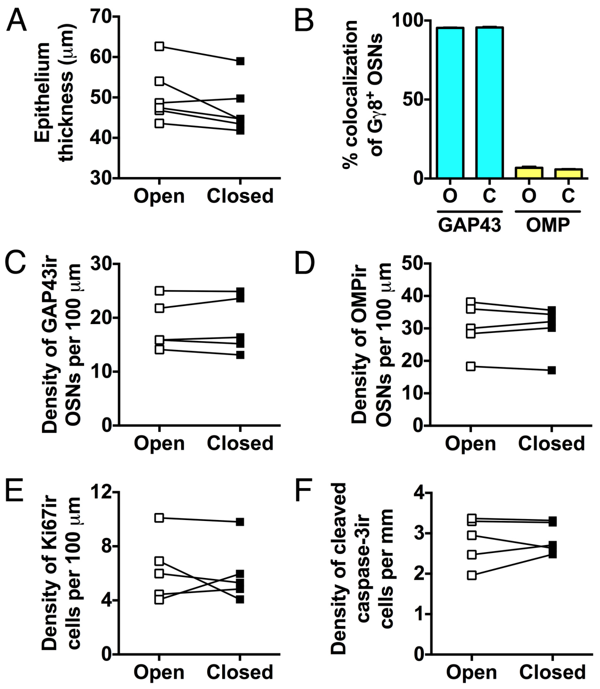

Supplementary Figure 5: Naris occlusion does not affect OSN numbers in the olfactory epithelium of Gγ8-sypGFP-tdTom or OMP-sypGFP-tdTom mice. All data is from 8 wk-old mice following three weeks of unilateral naris occlusion (right naris). **(A)** Tendency for thinner olfactory epithelium in the closed than the open naris (open: 50.5 ± 6.8 μm; closed 47.2 ± 6.4 μm; P = 0.066, paired t-test, n = 6 mice). Lines link data points for individual mice. **(B)** No effect of naris occlusion on colocalization of Gγ8-tdTom OSNs with either GAP43 (P = 0.62) or OMP (P = 0.17; paired t-tests, n = 3 mice). O: open naris, C: closed (occluded) naris. Data shown as mean ± s.d. **(C)** No effect of naris occlusion on density of GAP43-immunoreactive OSNs in the olfactory epithelium (P = 0.85, paired t-test, n = 5 mice). **(D)** No effect of naris occlusion on density of OMP-immunoreactive OSNs in the olfactory epithelium (P = 0.77, paired t-test, n = 5 mice). **(E)** No effect of naris occlusion on number of Ki67-immunoreactive proliferating cells in the olfactory epithelium (P = 0.71, paired t-test, n = 5 mice). **(F)** No effect of naris occlusion on number of cleaved caspase-3-immunoreactive apoptotic cells in the olfactory epithelium (P = 0.64, paired t-test, n = 5 mice).

OMP-tTA	Forward	GGTTGCGTATTGGAAGATCAAGAGC
	Reverse	GAGGAGCAGCTAGAAGAATGTCCC
Gγ8-tTA	Forward	GTTCCAGCCCCCAGTCCACACTCC
	Reverse	CATGTCCAGATCGAAATCGTCTAGC
CaMKIIα-tTA	Forward	CGCTGTGGGGCATTTTACTTTAG
	Reverse	CATGTCCAGATCGAAATC
tetO-sypGFP-tdTom<br>tetO-ChIEF-Citrine	Forward	GTTCATCTGCACCACCGGCAAGC
	Reverse	TGTGGCGGGTCTTGAAGTTCACC

Supplementary Table 1: Primers used for genotyping

Antigen	Species	Dilution	Supplier	Catalog No.
Cleaved caspase-3	Rabbit	1:200	Cell Signaling Technologies	Asp175
GAP43	Rabbit	1:1000	Novus Biologicals	NB300-143
GFP	Rabbit	1:1000	Clontech	632592
Ki67	Rabbit	1:200	Abcam	ab16667
OMP	Goat	1:5000	Wako	544-10001
PSD-95	Rabbit	1:250	Novus Biologicals	NBP1-40474
Synaptophysin	Mouse	1:500	Millipore	MAB5258
Synaptic vesicle glycoprotein 2A	Mouse	1:1600	DSHB	SV2 Concentrate
Tyrosine hydroxylase	Rabbit	1:2500	Novus Biologicals	NB300-109

Supplementary Table 2: Primary antibodies used for immunohistochemistry

	False positive rate (%)	False negative rate (%)	n (OMP-syp)<br>n (Gγ8-syp)
Anti-syp	3.8	n/a	116<br>123
Anti-PSD-95	11.3	n/a	122<br>116
Anti-GFP	0	1.3	120<br>119

Supplementary Table 3: False positive and false negative rates for anti-syp, anti-PSD-95 and anti-GFP immunostaining.

Note that the false negative rate for anti-syp staining could not be determined because syp is expressed in other OSN presynaptic terminals, and the false negative rate for anti-PSD-95 staining could not be determined because PSD-95 is also present at other synapses within the glomerulus. All analyzed puncta were in the glomerular layer. n = 3 Gγ8-sypGFP-tdTom and 3 OMP-sypGFP-tdTom mice.

References

1. De Paola, V. *et al.* Cell Type-Specific Structural Plasticity of Axonal Branches and Boutons in the Adult Neocortex. *Neuron* **49,** 861–875 (2006).
2. Grillo, F. W. *et al.* Increased axonal bouton dynamics in the aging mouse cortex. in E1514–E1523 (2013). doi:10.1073/pnas.1218731110/-/DCSupplemental
